# Development of *Staphylococcus* Enzybiotics: The Ph28 Gene of *Staphylococcus epidermidis* Phage PH15 Is a Two-Domain Endolysin

**DOI:** 10.3390/antibiotics9040148

**Published:** 2020-03-30

**Authors:** Magdy Mohamed Muharram, Ashraf Tawfik Abulhamd, Mohammed F. Aldawsari, Mohamed Hamed Alqarni, Nikolaos E. Labrou

**Affiliations:** 1 Department of Pharmaceutics, College of Pharmacy, Prince Sattam Bin Abdulaziz University, Alkharj 11942, Saudi Arabia; moh.aldawsari@psau.edu.sa; 2 Department of Microbiology, College of Science, Al-Azhar University, Cairo 11884, Egypt; Abulhamd@yahoo.com; 3 Department of Medical Laboratory Sciences, College of Applied Medical Sciences, Prince Sattam Bin AbdulAziz University, P.O. Box 173, Al-Kharj 11942, Saudi Arabia; 4 Department of Pharmacognosy, College of Pharmacy, Prince Sattam Bin Abdulaziz University, Alkharj 11942, Saudi Arabia; m.alqarni@psau.edu.sa; 5 Laboratory of Enzyme Technology, Department of Biotechnology, School of Food, Biotechnology and Development, Agricultural University of Athens, 75 Iera Odos Street, 11855 Athens, Greece; lambrou@aua.gr

**Keywords:** Endolysin, bacteriophage lysins, enzybiotics, multidrug-resistant, *Staphylococcus epidermidis*

## Abstract

Given the worldwide increase in antibiotic resistant bacteria, bacteriophage derived endolysins represent a very promising new alternative class of antibacterials in the fight against infectious diseases. Endolysins are able to degrade the prokaryotic cell wall, and therefore have potential to be exploited for biotechnological and medical purposes. *Staphylococcus epidermidis* is a Gram-positive multidrug-resistant (MDR) bacterium of human skin. It is a health concern as it is involved in nosocomial infections. Genome-based screening approach of the complete genome of *Staphylococcus* virus PH15 allowed the identification of an endolysin gene (Ph28; NCBI accession number: YP_950690). Bioinformatics analysis of the Ph28 protein predicted that it is a two-domain enzyme composed by a CHAP (22-112) and MurNAc-LAA (171-349) domain. Phylogenetic analysis and molecular modelling studies revealed the structural and evolutionary features of both domains. The MurNAc-LAA domain was cloned, and expressed in *E. coli* BL21 (DE3). In turbidity reduction assays, the recombinant enzyme can lyse more efficiently untreated *S. epidermidis* cells, compared to other *Staphylococcus* strains, suggesting enhanced specificity for *S. epidermidis*. These results suggest that the MurNAc-LAA domain from Ph28 endolysin may represent a promising new enzybiotic.

## 1. Introduction

The emergence of multidrug-resistant (MDR) bacteria is an extremely important threat to public health worldwide. The diversity of resistance mechanisms that contributes to the development of MDR may lead to pandrug resistance (PDR) [1,2]. Considering that the progress in discovering new antibiotics against MDR pathogens is very slow [3], it is conceivable that new strategies for controlling MDR or even PDR strains are urgently needed. This priority is currently being addressed by the use of multidisciplinary research efforts that aim to discovery new biomolecules able to effectively cope with the global emergence of MDR bacteria [4,5]. 

Peptidoglycan hydrolases (also known as enzybiotics, lytic enzymes, or endolysins) attract considerable interest as potential antibacterial tools [6]. These enzymes are responsible for digesting the bacterium cell wall. They are considered as excellent candidates for the development of novel therapeutics because they show a broad range of activity, are species-specific, possess high killing efficiency, are bactericidal (not just bacteriostatic), act in a short contact time, and they do not develop resistance [7,8,9]. Additional potential applications of enzybiotics include several scientific areas such as agricultural (e.g., treatment of phytopathogens) [10], veterinary (e.g., treatment of animal pathogens) [11], and for controlling bacterial contamination in the food industry (e.g., food-borne pathogens) [12]. 

Endolysins are large group of enzymes with diverse specificity towards the various bonds within the peptidoglycan (PG) [6,13,14]. For example, the polysaccharide backbone can be possessed by glycosyl hydrolases (muramidases/lysozymes and glucosaminidases), the initial L-alanine of the pentapeptide stem can be cleavaged by alanine amidases, and the subsequent peptide bonds in the stem or cross bridge can be modified by endopeptidases [6,14]. 

*S. epidermidis* is a Gram-positive commensal bacterium of the human skin microbiome [15]. The development of enzybiotics towards *S. epidermidis* is an important goal as it has emerged as a major nosocomial pathogen associated with infections of implanted medical devices [16]. Some *S. epidermidis* strains behave as pathogens colonizing surgery wounds and in some circumstances, they reach the human bloodstream causing severe bacteremia and potential mortality [17,18]. Children are especially prone to acquire methicillin-resistant *S. epidermidis* strains in perinatal hospitals [18,19]. In addition, *S. epidermidis* is thought to be associated with periodontitis, acute and chronic pulpitis, pericoronitis, dry socket and angular stomatitis [20].

In the present work, we identified and characterized an endolysin gene from the *Staphylococcus* virus PH15. The findings of the present study can be useful in the future for designing specific endolysins capable of coping with MDR *S. epidermidis* strains. 

## 2. Results and Discussion

### 2.1. The Ph28 Gene of S. Epidermidis Phage PH15 is a Two-domain Endolysin

The *Staphylococcus* phage PH15 (accession number NC_008723.1) is a dsDNA virus that belongs to the Siphoviridae family [21]. Phylogenetic analyses have showed that PH15 is clustered with Staphylococcus aureus and creates a novel clade within the S. aureus group [21]. The PH15 genome contained two introns, and in vivo splicing of phage mRNAs has been demonstrated for both introns [21].

**Figure 1:**
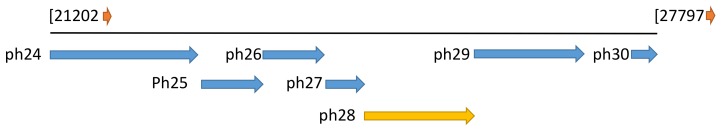
Genomic context of the ph28 gene in the *Staphylococcus* phage PH15 (accession number NC_008723.1). The gene of ph28 endolysin is located between 24765 to 26147 bp. The other neighbor proteins that appears on the figure are: ph24, putative hydrolase; ph25, hypothetical protein; ph26, hypothetical protein; ph27, putative phage holin; ph29: conserved phage protein; ph30: hypothetical protein.

**Figure 2:**
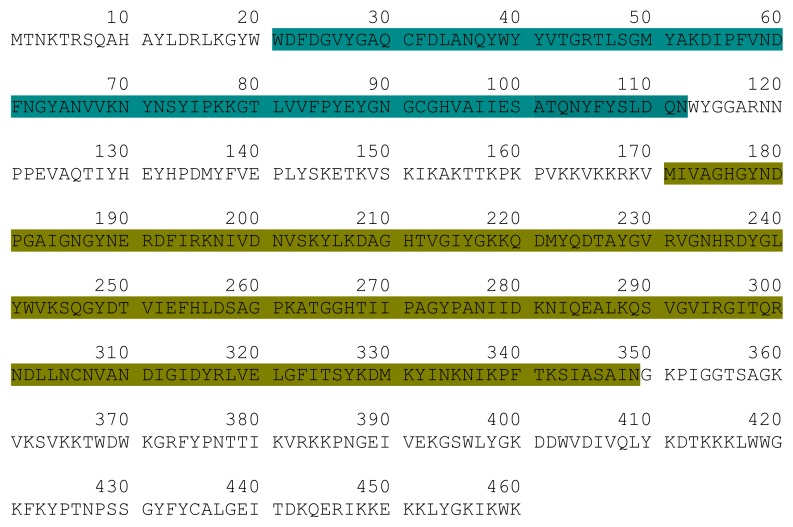
Schematic representation of the Ph28 endolysin sequence. The CHAP domain, is colored teal. The MurNac-LAA domain is colored dark yellow.

Genome-based screening of *Staphylococcus* phage PH15 allowed us the identification of a single putative endolysin (Ph28 gene; accession number: YP_950690) in its genome. The Ph28 endolysin is located between 24,765 to 26,147 bp (Figure 1). It is composed by 460 amino acids (Figure 2) with predicted molecular mass 52,620.06 Da and theoretic isoelectric point (pI) 9.54. 

**Figure 3:**
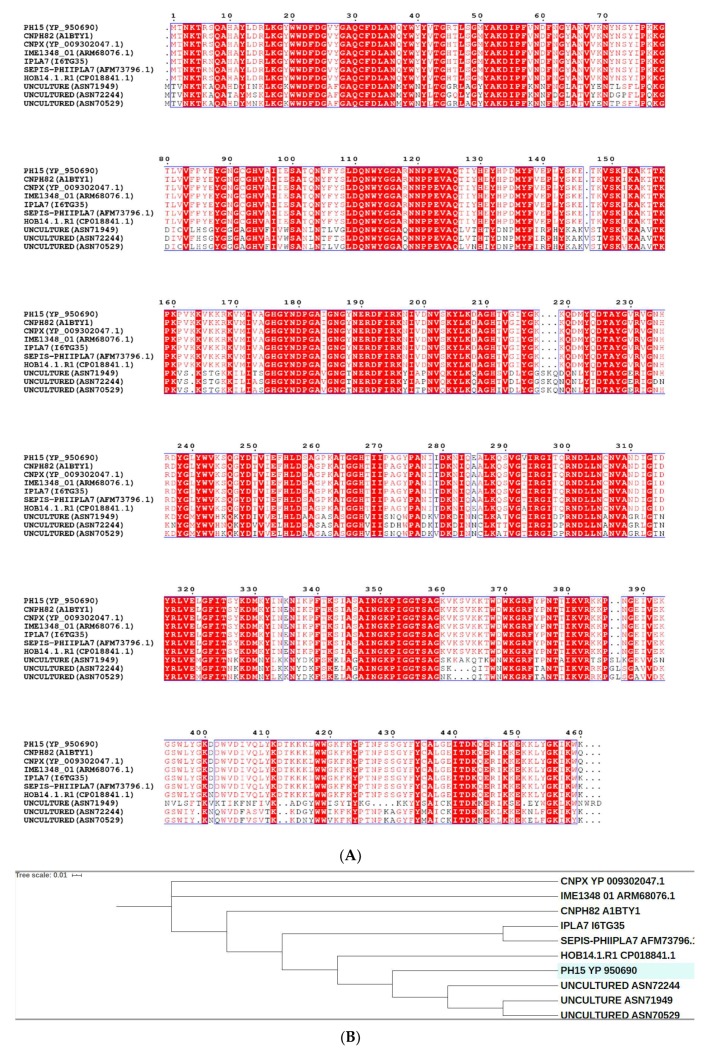
(**A**). Amino acid sequence alignements of endolysin Ph28 with other putative endolysin sequences from *Staphylococcus* viruses that infect *S. epidermidis* strains. The alignments were produced using Clustal Omega [26]. Conserved areas are shown shaded. A column is framed, if more than 70% of its residues are similar according to physico-chemical properties. (**B**). Phylogenetic analysis of endolysin Ph28. Phylogenetic tree was constructed by Neighbour-Joining method using the iTOL programme [27]. The tree was formed after alignment of the protein sequences using Clustal Omega [26]. The endolysin sequences were derived from: *Staphylococcus* virus CNPH82 (NCBI accession number A1BTY1), *Staphylococcus* virus IPLA7 (NCBI accession number I6TG35), *Staphylococcus* phage CNPx (NCBI accession number YP_009302047.1), *Staphylococcus* phage vB_SepiS-phiIPLA7 (AFM73796.1), *Staphylococcus* phage IME1348_(NCBI accession number ARM68076.1), uncultured Caudovirales phage (NCBI accession number ASN72244), uncultured Caudovirales phage (NCBI accession number ASN70529), uncultured Caudovirales phage (NCBI accession number ASN71949), *Staphylococcus* phage HOB 14.1.R1 (NCBI accession number CP018841.1).

The amino acid sequence of Ph28 endolysin was analyzed in silico, aiming at elucidating its domain architecture. Conserved sequence patterns observed between Ph28 endolysin and a set of recently sequenced *Staphylococcus* phages endolysins were identified using the Conserved Domains Database (CDD) (Figure 2 and Figure 3) [22]. The analysis showed that the Ph28 endolysin is a two-domain enzyme composed of a cysteine, histidine-dependent amidohydrolases/peptidase (CHAP, pfam05257) (aa 22-112) and a N-acetylmuramoyl-L-alanine amidase (MurNAc-LAA, cd02696) (aa 171-349) domains. Endolysins with this domain type were encoded by phage families Siphoviridae, Podoviridae, and Myoviridae that infect bacterial genera *Staphylococcus* and *Streptococcus*. Amino acid sequence alignements of the endolysin Ph28 with other putative endolysin sequences from *Staphylococcus* viruses that infect S. epidermidis strains (Figure 3) revealed a higher degree of identity between the CHAP domains, compared with the catalytic MurNac-LAA domain. For example, the identity between the different MurNac-LAA domains ranges between 56% and 98%, whereas between the different the CHAP domains ranges between 65% and 99%. This observation indicates that the CHAP and MurNac-LAA domains have evolved under differential selective pressures. The lower degree of identity observed for the catalytic MurNac-LAA domain suggests higher structural diversity that probably reflects differences in catalysis and substrate specificity. To understand the relation between the selected endolysins, a phylogenetic analysis was performed (Figure 3B). For that, a cladogram was constructed using putative endolysin sequences derived from *Staphylococcus* viruses that infect S. epidermidis strains. The ten enzymes were clustered into three separate clades. The first contains the *Staphylococcus* phage CNPx, the second the *Staphylococcus* phage IME1348, and the third clade includes all other endolysin sequences. The endolysin Ph28 is closer to putative endolysins that have been isolated from uncultured human skin metaviromes (uncultured Caudovirales phage ASN72244, ASN70529, and ASN71949) than to other staphylococcal endolysins.

In general, phage endolysins that infect Gram-positive bacteria, such as *S. epidermidis*, display a modular architecture. They are composed by an enzymatic catalytic domain (ECD), located at the N-terminal part of the protein, and at least one cell wall binding domain (CBD), which is located at the C-terminal part. The ECD and CBD domains are linked by a flexible interdomain polypeptide [9]. Phage endolysins that infect Gram-negative bacteria show a more globular architecture, composed by a single catalytic domain [23]. Therefore, the protein families of endolysin domains are in dependence of bacterial host Gram-type. In particular, domains belonging to lysozyme-like family (cl00222, cd00442), bacteriophage lambda lysozyme domain family (cd00736), and endolysin/autolysin domain family (cd00737) represent typical domains for Gram-negative bacteria. On the other hand, for the Gram-positive bacteria, domains belonging to GH25 muramidase 1 (cd06413) family and alanine amidases belonging to superfamily MurNAc-LAA (cl02713) are included [24,25].

In bacteria, the MurNAc-LAA domain belongs to the autolysin system that hydrolyzes the amide bond between N-acetylmuramoyl and L-amino acids in certain cell wall glycopeptides. In general, bacterial MurNAc-LAAs carry a signal peptide in their N-termini that allows their transport across the cytoplasmic membrane. In contrast, MurNAc-LAAs from bacteriophages are endolysins since they are able to break down bacterial peptidoglycan at the terminal stage of the phage reproduction cycle. 

All phage-encoded endolysins have no signal peptides and their translocation through the cytoplasmic membrane is supported by the phage-encoded holin proteins [25].

The CHAP domain (pfam05257) is a polypeptide composed by 110 to 140 amino acids that is found in proteins from bacteria, bacteriophages, archaea and eukaryotes of the Trypanosomidae family. The CHAP domain adopts a wide range of architectures and it is usually connected with bacterial type SH3 domains or with several families of amidase domains [24]. 

### 2.2. Molecular Modelling and Structural Analysis

**Figure 4:**
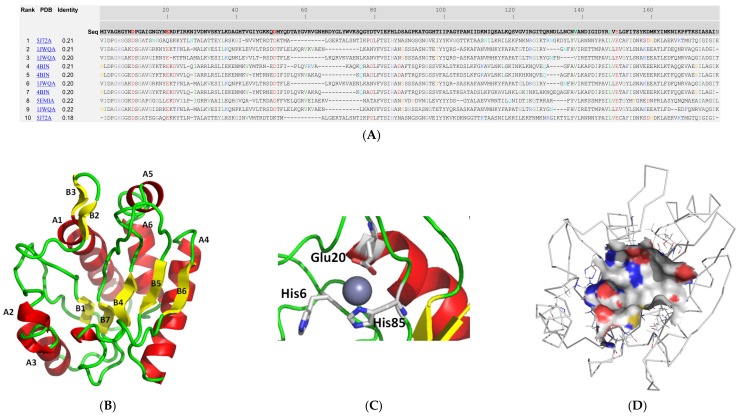
Structure prediction of the MurNac-LAA domain using the I-TASSER approach [28,29]. (**A**). Ten best threading templates that were used by I-TASSER. Templates were identified by LOMETS threading programs [32] from the PDB library. All the residues are colored in black; however, those residues in template which are identical to the residue in the query sequence are highlighted in color. Coloring scheme is based on the property of amino acids, where polar are brightly coloured while non-polar residues are colored in dark shade. The column ‘identity’ is the percentage sequence identity of the templates in the threading aligned region with the query sequence. The top 10 alignments reported above (in order of their ranking) are from the following threading programs: 1, MUSTER; 2, FFAS-3D; 3, SPARKS-X; 4, HHSEARCH2; 5, HHSEARCH I; 6, Neff-PPAS; 7, HHSEARCH; 8, pGenTHREADER; 9, PROSPECT2; 10, PRC. (**B**): Ribbon diagram of MurNac-LAA domain. α-Helices are colored red and β-strands yellow. (**C**). The predicted zinc binding residues. Zinc is shown as a sphere (**D**). Surface aria of the predicted ligand binding site. The drawings were created using PyMOL.

**Figure 5:**
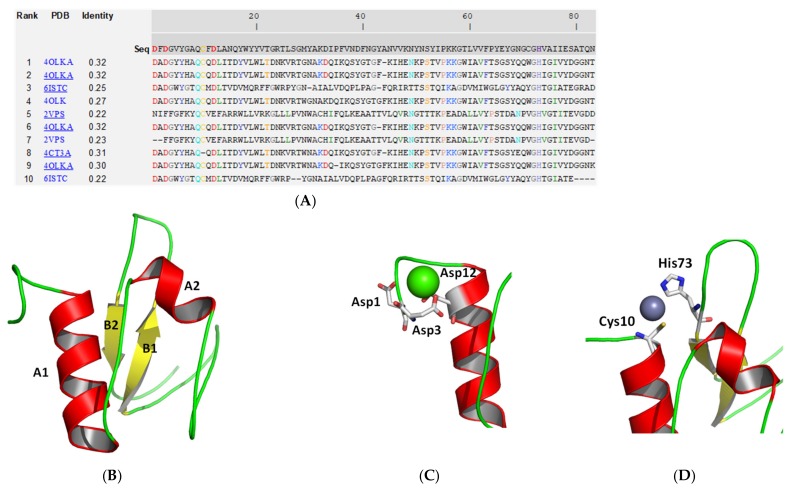
Structure prediction of the CHAP domain using the I-TASSER approach [26,27]. (**A**). Ten best threading templates that were used by I-TASSER. Templates were identified by LOMETS threading programs [32] from the PDB library. Conserved residues are colored. Coloring scheme is based on the property of amino acids, where polar are brightly colored while non-polar residues are colored in dark shade. The column ‘identity’ is the percentage sequence identity of the templates in the threading aligned region with the query sequence. The top 10 alignments reported above (in order of their ranking) are from the following threading programs: 1, MUSTER; 2, FFAS-3D; 3, SPARKS-X; 4, HHSEARCH2; 5, HHSEARCH I; 6, Neff-PPAS; 7, HHSEARCH; 8, pGenTHREADER; 9, PROSPECT2; 10, PRC. (**B**): Ribbon diagram of CHAP domain. α-Helices are colored red and β-strands yellow. (**C**). The predicted calcium binding residues. Calcium is shown as a sphere. (**D**). The predicted zinc binding residues. Zinc is shown as a sphere. The drawings were created using PyMOL.

To understand the structural properties of the MurNAc-LAA and CHAP domains protein, their 3D structures were predicted by the I-TASSER (iterative threading assembly refinement) approach [28,29]. The two domains were modelled separate as there is no available template in the PDB for the entire endolysin sequence (aa 1-460). The location of the ligand-binding site was predicted by COFACTOR [30] and COACH [31] based on the I-TASSER structure prediction. As shown in Figure 4 and Figure 5, both domains display distinct structural elements and fold into α/β structures. The MurNAc-LAA domain is composed by six helices and six beta strands (Figure 4B). The linker peptide that connects the two domains is located at the end of α-helix A6 of the MurNAc-LAA domain. The location of the substrate binding site of the MurNAc-LAA domain was predicted at an exposed cleft between the A1 and A2 helices. It is formed by different sequence fragments that are located in separate regions of the primary structure. A zinc ion was predicted in the cleft, bound to three conserved amino acids His6, Glu20, and His85 (Figure 4C). Other residues that form the ligand binding region are: Pro101, Asn137, Val138, Ala139, Asn140, Asp141, Leu148, Glu150 (Figure 4D), suggesting that non-polar as well as negatively charged residues are important structural elements for ligand binding. Among them, Val138, Leu148 and Glu150 are conserved in all homologue sequences (Figure 4A). 

The CHAP domain composed by two helices and its C-terminal is composed by two beta strands (Figure 5B). Proteins containing CHAP domain, use a cysteine residue in the catalytic mechanism of the nucleophilic attack [24,25]. The CHAP domain of endolysin Ph28 contains the conserved Cys10 and His73 as residues that interact with a zinc ion and form the putative ligand binding region of the protein. Other residues that contribute to the formation of the ligand binding region are Gln9, Ala31, Cys71, Val74, which are only partially conserved in the homologue sequences (Figure 5A). In addition, a calcium ion is predicted at the N-terminal part, bound to the three conserved aspartic acid residues (Asp1, Asp3 and Asp12). 

**Figure 6:**
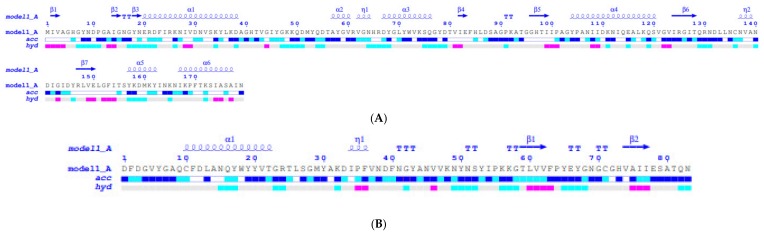
ENDscript analysis [33]. Flat figure showing the sequence of the MurNac-LAA (**A**) and CHAP (**B**) models, predicted by the I-TASSER approach, with secondary structure elements presented on top (helices with squiggles, β-strands with arrows and turns with TT letters). Solvent accessibility is rendered by a first bar below the sequence (blue is accessible, cyan is intermediate, white is buried) and hydropathy by a second bar below (pink is hydrophobic, white is neutral, cyan is hydrophilic)

Solvent accessibility and hydropathy analysis of the MurNAc-LAA and CHAP domains suggest that their ligand binding sites are accessible, exposed to the solvent and formed mainly by hydrophilic and neutral aminoacid residues (Figure 6).

### 2.3. Turbidity Reduction Assay of Recombinant Ph28 Endolysin 

**Figure 7:**
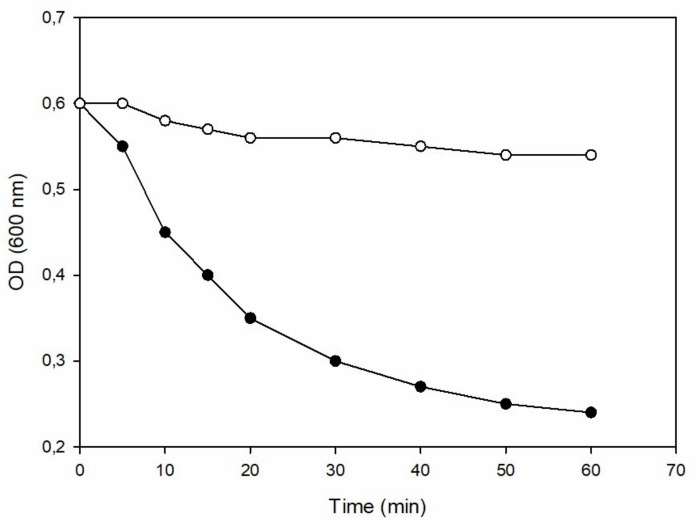
Enzyme activity of the recombinant MurNac-LAA domain. Enzyme activity was demonstrated by measuring the time-dependent turbidity changes in a suspension of *S. epidermidis* (ATCC 12228) cells. Changes in turbidity was measured spectrophotometrically at 600 nm at regular time intervals and at a fixed temperature (25 °C), using extract from recombinant *E. coli* BL21(DE3) cells (●), compared to a negative control using untransformed *E. coli* BL21(DE3) cells (○).

To demonstrate that ph28 gene codes for an active endolysin, the full-length protein as well as the MurNac-LAA domain were cloned and expressed in *E. coli*. The full-length protein could not be expressed in soluble or insoluble form in several different *E. coli* strains [C41(DE3), C43(DE3), BL21(DE3), or BL21(DE3) pLysS] and under different culture conditions (37 °C, 25 °C, 18 °C; 0.1–1 mM IPTG; LB medium or YT medium). On the other hand, the MurNac-LAA domain was expressed in *E. coli* BL21(DE3) and its catalytic activity and specificity was evaluated. The most common assay for determining the endolysins activity is based on the drop in optical density of the substrate after addition of the enzyme [5,34,35]. Thus, the activity of MurNac-LAA was determined using a turbidity reduction assay from time-dependent turbidity changes, (OD600 nm) versus time, in a suspension of *S. epidermidis* cells, as shown in Figure 7. The results show that the MurNac-LAA domain is able to reduce the turbidity of the MurNac-LAA cell suspension, suggesting an active lytic enzyme.

The specificity of the catalytic MurNac-LAA domain was further assessed using a range of different *S. epidermidis* strains (ATCC 12228, ATCC 14990, ATCC 700576) as well as different Staphylococcus species and the results are listed in Table 1. The results showed that the MurNac-LAA domain exhibits comparable lytic activity towards different *S. epidermidis* strains. On the other hand, it displays higher lytic activity towards *S. epidermidis* strains, compared to other *Staphylococcus* species (e.g. *Staphylococcus caprae*, *Staphylococcus capitis, Staphylococcus haemolyticus*), suggesting a higher preference and presumably enhanced specificity for *S. epidermidis*.

## 3. Materials and Methods 

### 3.1. Biocomputing Analysis

Aminoacid sequence alignments were carried out using Clustal O [26]. Retrieval of protein sequences and domain prediction were accomplished by BLASTp [36]. ESPript 3.0 and ENDscript [30] were employed for the analysis and visualization of sequence alignments. The molecular models of MurNAc-LAA and CHAP domains were predicted using the I-TASSER (iterative threading assembly refinement) approach [28,29]. The confidence of the models generated by I-TASSER was quantitatively measured and assessed by C-score that is calculated based on the significance of threading template alignments and the convergence parameters of the structure assembly simulations [28,29]. C-score is typically in the range of [-5 to 2], where a C-score of higher value signifies a model with a high confidence and *vice-versa*. The C-score for the MurNAc-LAA and CHAP domain were 0.35 and 0.38, respectively, suggesting the validity of both models [28,29]. The location of the ligand-binding site was predicted by COFACTOR [30] and COACH [31] based on the I-TASSER structure prediction. The molecular model of the MurNAc-LAA domain in complex with Zn^+2^ was predicted based on PDB structure 3QAYA. The molecular model of the CHAP domain in complex with Ca^+2^ was predicted based on PDB structure 4OLKA. The program PyMOL (www.pymol.org) was used for structures visualization. 

### 3.2. Cloning and Expression of the MurNAc-LAA Domain

The nucleotide sequence encoding the MurNAc-LAA domain (aa171-349) was synthesized and amplified by PCR. The PCR reaction was carried out in a total volume of 50 μL, containing 8 pmoles of each primer: 5’- ATGACAAACAAAACGAGAAGTC-3’ and 5’- TTAATTAATAGCGCTTGCTATTGACTTTGT-3’ 10 ng template DNA, 0.5 mM dNTPs, 25 μL 2× HF reaction buffer and 1U Pfu DNA polymerase. The PCR-primers were designed according to the Ph28 gene sequence. The PCR protocol was composed by an initial denaturation at 94 °C for 30 sec, then 25 cycles of 15 sec at 94 °C, 15 sec at 65 °C and 1 min at 72 °C. A final extension of time at 72 °C for 10 min was performed after the 25th cycle. The PCR product was cloned to the pEXP5-CT/TOPO-TA vector (Invitrogen).

### 3.3. Expression of MurNAc-LAA Domain in E. coli BL21(DE3)

Transformed *E. coli* BL21(DE3) cells with the recombinant plasmid were grown at 37 °C in 1 L LB medium containing ampicillin (100 μg/mL). When the absorbance of the culture at 600 nm was 0.4 AU, the expression of MurNAc-LAA domain was induced by the addition of 1 mM isopropyl 1-thio-β-galactopyranoside (IPTG). Following five hours incubation, the culture was centrifuged at 10,000 rpm for 10 min. The cell pellet was resuspended in potassium phosphate buffer (20 mM, pH 7) and sonicated. Cell debris were removed by centrifugation at 13,000 rpm for 5 min.

### 3.4. Assay of Enzyme Activity

The in vitro assessment of peptidoglycan-degrading activity of MurNAc-LAA domain was performed using the turbidimetry assay as described previously [5,31,32]. Enzyme lytic activity (rate constant, min^−1^) was determined by fitting the single exponential decay equation to the experimental data (absorbance). *E. coli* BL21(DE3) cells were used as negative control. 

## 4. Conclusions

In conclusion, phages have evolved several classes of endolysins aiming at degrading the cell wall of their bacterial hosts. Although endolysins have a common enzymatic activity (e.g., the hydrolysis of cell wall), several distinct classes exist with different catalytic mechanisms, structures, and domain architectures. The *S. epidermidis* bacteriophage PH15 endolysin is a two domain protein (CHAP and MurNAc-LAA). The the MurNAc-LAA domain was cloned, expressed in *E. coli* BL21 (DE3), and its ability to catalyze the lysis of *S. epidermidis* was demonstrated. These results suggest that the MurNAc-LAA from *Staphylococcus* phage PH15 may represent a potential new enzybiotic. 

## Figures and Tables

**Table 1:** Lytic activity of the catalytic MurNac-LAA domain towards different *S. epidermidis* strains and *Staphylococcus* species.

Strains/Species	Rate Constant (min^−1^)
*S. epidermidis* (ATCC 12228)	0.052 ± 0.005
*S. epidermidis* (ATCC 14990)	0.053 ± 0.004
*S. epidermidis* (ATCC 700576)	0.055 ± 0.004
*S. caprae* (ATCC 55133)	0.032 ± 0.002
*S. capitis* (ATCC 146 )	0.029 ± 0.002
*S. haemolyticus* (ATCC 31874 )	0.026 ± 0.003
*E. coli* BL21(DE3)	0.004 ± 0.002

## References

[1] Potron A., Poirel L., Nordmann P. (2015). Emerging broad-spectrum resistance in pseudomonas aeruginosa and Acinetobacter baumannii: Mechanisms and epidemiology. Int. J. Antimicrob. Agents.

[2] Ventola C.L. (2015). The antibiotic resistance crisis: Part 2: Management strategies and new agents. Pharm. Ther..

[3] Fischbach M.A., Walsh C.T. (2009). Antibiotics for emerging pathogens. Science.

[4] Briers Y., Lavigne R. (2015). Breaking barriers: Expansion of the use of endolysins as novel antibacterials against Gram-negative bacteria. Future Microbiol..

[5] Oliveira H., São-José C., Azeredo J. (2018). Phage-derived peptidoglycan degrading enzymes: Challenges and future prospects for in vivo therapy. Viruses.

[6] Alcorlo M., Martínez-Caballero S., Molina R., Hermoso J.A. (2017). Carbohydrate recognition and lysis by bacterial peptidoglycan hydrolases. Curr. Opin. Struct. Biol..

[7] Gerstmans H., Criel B., Briers Y. (2018). Synthetic biology of modular endolysins. Biotechnol Adv..

[8] Abdelkader K., Gerstmans H., Saafan A., Dishisha T., Briers Y. (2019). The preclinical and clinical progress of bacteriophages and their lytic enzymes: The parts are easier than the whole. Viruses.

[9] Dams D., Briers Y. (2019). Enzybiotics: Enzyme-based antibacterials as therapeutics. Adv. Exp. Med. Biol..

[10] Mansfield J., Genin S., Magori S., Citovsky V., Sriariyanum M., Ronald P., Dow M.A., Verdier V., Beer S.V., Machado M.A. (2012). Top 10 plant pathogenic bacteria in molecular plant pathology. Mol. Plant Pathol..

[11] Fischetti V.A. (2010). Bacteriophage endolysins: A novel anti-infective to control gram-positive pathogens. Int. J. Med Microbiol..

[12] Schmelcher M., Loessner M.J. (2016). Bacteriophage endolysins: Applications for food safety. Curr. Opin. Biotechnol..

[13] Vidová B., Šramková Z., Tišáková L., Oravkinová M., Godány A. (2014). Bioinformatics analysis of bacteriophage and prophage endolysin domains. Biologia.

[14] Wittekind M., Schuch R. (2016). Cell wall hydrolases and antibiotics: Exploiting synergy to create efficacious new antimicrobial treatments. Curr. Opin. Microbiol..

[15] Byrd A.L., Belkaid Y., Segre J.A. (2018). The human skin microbiome. Nat. Rev. Microbiol..

[16] Lim S.M., Webb S.A. (2005). Nosocomial bacterial infections in intensive care units. I: Organisms and mechanisms of antibiotic resistance. Anaesthesia.

[17] Chessa D., Ganau G., Mazzarello V. (2015). An overview of *Staphylococcus epidermidis* and *Staphylococcus aureus* with a focus on developing countries. J. Infect. Dev. Ctries..

[18] Ortega-Peña S., Martínez-García S., Rodríguez-Martínez S., Cancino-Diaz M.E., Cancino-Diaz J.C. (2020). Overview of *Staphylococcus epidermidis* cell wall-anchored proteins: Potential targets to inhibit biofilm formation. Mol. Biol. Rep..

[19] Cabrera-Contreras R., Santamaría R.I., Bustos P., Martínez-Flores I., Meléndez-Herrada E., Morelos-Ramírez R., Barbosa-Amezcua M., González-Covarrubias V., Silva-Herzog E., Soberón X. (2019). Genomic diversity of prevalent *Staphylococcus epidermidis* multidrug-resistant strains isolated from a Children’s Hospital in México City in an eight-years survey. PeerJ.

[20] Namvar A.E., Bastarahang S., Abbasi N., Ghehi G.S., Farhadbakhtiarian S., Arezi P., Hosseini M., Baravati S.Z., Jokar Z., Chermahin S.G. (2014). Clinical characteristics of *Staphylococcus epidermidis*: A systematic review. GMS Hyg. Infect. Control.

[21] Daniel A., Bonnen P.E., Fischetti V.A. (2007). First complete genome sequence of two *Staphylococcus epidermidis* bacteriophages. J. Bacteriol..

[22] Marchant E.A., Boyce G.K., Sadarangani M., Lavoie P.M. (2013). Neonatal sepsis due to coagulase-negative staphylococci. Clin. Dev. Immunol..

[23] Pohane A.A., and Jain V. (2015). Insights into the regulation of bacteriophage endolysin: Multiple means to the same end. Microbiology.

[24] Bateman A., Rawlings N.D. (2003). The CHAP domain: A large family of amidases including GSP amidase and peptidoglycan hydrolases. Trends Biochem. Sci..

[25] Rigden D.J., Jedrzejas M.J., Galperin M.Y. (2003). Amidase domains from bacterial and phage autolysins define a family of gamma-D,L-glutamate-specific amidohydrolases. Trends Biochem. Sci..

[26] Sievers F., Wilm A., Dineen D., Gibson T.J., Karplus K., Li W., Lopez R., McWilliam H., Remmert M., Söding J. (2011). Fast, scalable generation of high-quality protein multiple sequence alignments using Clustal Omega. Mol. Syst. Biol..

[27] Letunic I., Bork P. (2019). Interactive Tree Of Life (iTOL) v4: Recent updates and new developments. Nucleic Acids Res..

[28] Roy A., Kucukural A., Zhang Y. (2010). I-TASSER: A unified platform for automated protein structure and function prediction. Nat. Protoc..

[29] Yang J., Yan R., Roy A., Xu D., Poisson J., Zhang Y. (2015). The I-TASSER Suite: Protein structure and function prediction. Nat. Methods.

[30] Zhang C., Freddolino P.L., Zhang Y. (2017). COFACTOR: Improved protein function prediction by combining structure, sequence and protein-protein interaction information. Nucleic Acids Res..

[31] Yang J., Roy A., Zhang Y. (2013). Protein-ligand binding site recognition using complementary binding-specific substructure comparison and sequence profile alignment. Bioinformatics.

[32] Zheng W., Zhang C., Wuyun Q., Pearce R., Li Y., Zhang Y. (2019). LOMETS2: Improved meta-threading server for fold-recognition and structure-based function annotation for distant-homology proteins. Nucleic Acids Res..

[33] Robert X., Gouet P. (2014). Deciphering key features in protein structures with the new ENDscript server. Nucleic Acids Res..

[34] Briers Y., Lavigne R., Volckaert G., Hertveldt K. (2007). A standardized approach for accurate quantification of murein hydrolase activity in high-throughput assays. J. Biochem. Biophys. Methods.

[35] Filatova L.Y., Donovan D.M., Foster-Frey J., Pugachev V.G., Dmitrieva N.F., Chubar T.A., Klyachko N.L., Kabanov A.V. (2015). Bacteriophage phi11 lysin: Physicochemical characterization and comparison with phage phi80α lysin. Enzym. Microb. Technol..

[36] Altschul S.F., Gish W., Miller W., Myers E.W., Lipman D.J. (1990). Basic local alignment search tool. J. Mol. Biol..

